# Context and Time in Causal Learning: Contingency and Mood Dependent Effects

**DOI:** 10.1371/journal.pone.0064063

**Published:** 2013-05-15

**Authors:** Rachel M. Msetfi, Caroline Wade, Robin A. Murphy

**Affiliations:** 1 Department of Psychology, University of Limerick, Limerick, Ireland; 2 Department of Experimental Psychology, University of Oxford, Oxford, United Kingdom; Peking University, China

## Abstract

Defining cues for instrumental causality are the temporal, spatial and contingency relationships between actions and their effects. In this study, we carried out a series of causal learning experiments that systematically manipulated time and context in positive and negative contingency conditions. In addition, we tested participants categorized as non-dysphoric and mildly dysphoric because depressed mood has been shown to affect the processing of all these causal cues. Findings showed that causal judgements made by non-dysphoric participants were contextualized at baseline and were affected by the temporal spacing of actions and effects only with generative, but not preventative, contingency relationships. Participants categorized as dysphoric made less contextualized causal ratings at baseline but were more sensitive than others to temporal manipulations across the contingencies. These effects were consistent with depression affecting causal learning through the effects of slowed time experience on accrued exposure to the context in which causal events took place. Taken together, these findings are consistent with associative approaches to causal judgement.

## Introduction

The ability to learn about causal relationships between events is adaptive and enables people to learn to control their environment or, at least, to interact with it effectively [1]. It isn't surprising then that psychological disturbance can affect people's judgments about causal relationships [2], [3]. Moreover, the underlying mechanisms through which such effects occur are of considerable interest, not least because they can inform about the psychological disturbance itself but also because they can inform us about the nature of causal learning processes. For example, the link between cause and effect is not directly observable and so causal learning involves a psychological process that extracts cues to causality, the temporal and contingency relationships between events [4], [5]. For example, cause and effect occur successively, often in close spatial proximity [6], and the effect should be contiguous and contingent upon the occurrence of the cause [7], [8]. However, it is the combination of these cues that is critical in terms of defining causality, as any of them taken in isolation could be misleading [9], [10].

Time, in terms of succession and contiguity, is often considered to be the essential causal cue, while patterns of cause-effect co-occurrence provide additional corroborative information [9], [11], [12]. In other words, if information about the contingency between cause and effect is consistent with a causal relationship, but time information is not, then people are less likely to judge that a causal relationship exists [7]. However, determining whether or not this is the case is complicated because there is considerable inter-connectedness between these variables. Time, for example, not only defines succession and contiguity [4] but also the density or rate of cause-effect experiences which is relevant to contingency [13]. Spatial proximity is defined by the context in which events occur, and the context itself can define action-effect contingency [14]. Moreover, the passage of time can constitute a change of context [15] and context is not a discretely occurring countable event, but a continuous and sometimes temporally defined aspect of a causal learning task [16]. Thus, it can be argued that time and contingency, and space or context and contingency, are inter-dependent and in studies so far, context was not a variable that was not manipulated and measured explicitly.

Our strategy in the current paper is to consider context explicitly, alongside the other causal cues, and to manipulate time and context systematically across different contingency conditions. Furthermore, we will also test distinct groups of participants categorized by levels of depressed mood. This is useful because depression is not only associated with changes in causal sensitivity [16], [17], [18], [19] but also with disturbed time perception [20], [21], [22] and impaired context processing [16], [23]. Therefore, levels of depression will have specific effects on causal judgments, and moderate the effects of time and context manipulations.

Our starting point is a brief discussion on the status of the various causal cues in relation to current theoretical perspectives on causal judgment, before we then discuss how studying the effects of depressed mood on causal judgment might be a useful method of informing this debate.

### Contingency and time in causal learning

Causal judgments often relate to contingencies with which people are actively involved rather than being passive observers and a considerable body of research has focused on whether judgments of such action-outcome contingencies bear any relation to objective mathematical quantifiers of the same relationships. Given the importance of contingency in causality, we will frame our arguments around this literature.

The contingency between an action and outcome can be quantified by a normative metric, known as delta P or *ΔP* [24]. *ΔP* is a value, similar to a correlation coefficient, which describes both the direction (generative, preventative) and strength (strong, weak) of a contingency relationship. It differs from the correlation coefficient in two ways in that it concerns the frequency of binary events (on/off) and is a measure of a one-way, rather than bidirectional, relationship. *ΔP* is calculated as the difference between the conditional probabilities of an outcome given the presence of an action, *p*(Outcome|Action), and the absence of an action, *p*(Outcome|No Action). Therefore, the calculated value can vary between +1, indicating a perfect positive contingency and a generative causal relationship between action and effect, through the continuum to -1, where the outcome is less likely to happen in the presence of the action than in its absence, a preventative negative contingency. In a situation where the conditional probabilities are equal, the effect is no more likely to occur in the presence of the outcome than in its absence, and the *ΔP* is zero. In other words, there is no contingency relationship between action and outcome.

**Figure 1:**
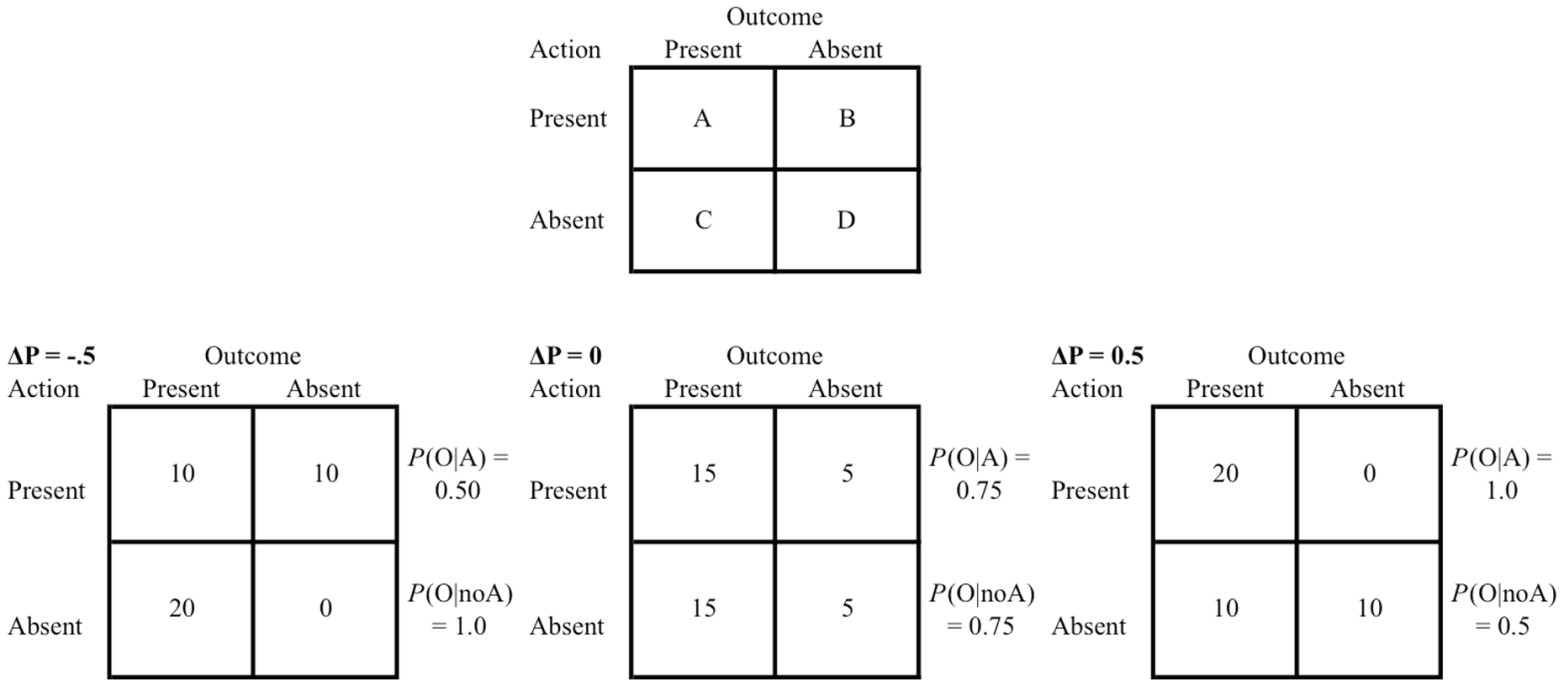
2×2 contingency tables showing the four possible combinations of action – effect information. Note: The upper table shows generic information from which *P* is calculated, where A, B, C and D refer to the frequencies of action – effect conjunctions. *ΔP* = A/(A+B) – C/(C+D). The lower tables show examples of three contingency conditions with a *ΔP* of −.5, 0 and +.5 respectively (left to right). P(O|A) refers to the conditional probability of the outcome given the presence of the action and P(O|noA) refers to the conditional probability of the outcome given the absence of the action.

This definition of contingency includes the assumption that four possible action-effect conjunctions are relevant to the *ΔP* calculations. Figure 1 (upper panel) shows a standardized contingency matrix in which the frequencies of each conjunction are given in each cell and denoted by the letters A, B, C and D. In many experimental designs, each conjunction would take place during a discrete experimental trial and the experimenter could manipulate the cell frequencies and thus the contingency experienced by the participant. Figure 1 (lower panel) shows examples of such manipulations, a negative contingency (left), a zero contingency (middle) and a positive contingency (right) condition. After being exposed to such conditions, participants would be asked to assess the contingency, perhaps by rating their degree of control over the effect on a numeric scale, which could then be compared to systematic manipulations of *ΔP*, similar to those shown in Figure 1.

Studies have shown that people are very sensitive to changes in manipulated contingencies. Their ratings of the strength of causal relationships distinguish between conditions, like those shown in Figure 1, in which effects are and are not contingent upon their actions [25], [26], [27]. In addition, ratings are highly correlated with much more subtle variations in *ΔP*. For example, Wasserman et al., [25] exposed their participants to 25 different conditions that involved subtle variations in contingency. Ratings were almost perfectly positively correlated with *ΔP* (*r* = .97), demonstrating remarkable isomorphism between causal ratings and variations in contingency. Notably, causal ratings were weakest in conditions in which contingency was zero (*ΔP* = 0), in spite of the fact that these conditions did involve contiguous pairings between actions and outcomes. Contingency, it seems, was a crucial cue to causality.

In spite of the importance of contingency for causal learning, temporal contiguity does have a profound effect on people's ability to detect causal relationships. For example, Shanks, Pearson and Dickinson [7] exposed their participants to positive contingencies in which the *ΔP* was .75 and the temporal delay between actions and outcomes varied from 0s to 16 s. A 2 s delay significantly reduced causal ratings and delays of greater than 4s completely eliminated any perception of causality. Thus, even in conditions with a strong contingency between action and outcome, degrading temporal contiguity attenuated and even eliminated the perception of causality. These and other findings suggested that time was a more critical cue to causality than contingency because, even in the case of a strong contingency, changing the temporal parameters of the task eliminated the perception of cause [7], [8], [28]. That being said, two assumptions underlie that conclusion, that the **experienced** **temporal sequence** of event-outcome conjunctions is consistent with that programmed by the experimenter, and thus, the **contingency experienced** by the participant is also isomorphic with that programmed by the experimenter. As we discuss later on in this paper, and has been discussed elsewhere [29], these are two assumptions which can be questioned.

### Theoretical Approaches to Causal Learning

The debate on whether time or contingency is the more critical or important causal cue relates to the two broad theoretical frameworks that each use different explanatory mechanisms to account for the effects of time and contingency on causal learning. One approach explains causal learning through a simple time-sensitive error correction learning algorithm and the development of simple associations between actions and outcomes, such that the strength of the association is correlated with the strength of the causal relationship [30]. Another theoretical approach holds that people use causal knowledge, including knowledge of the role of time in causality, to generate propositions or inferences about whether causality is present, before using contingency information to assess the strength of that relationship [11], [12], [31], [32]. Consistent with the latter view, situation specific knowledge of temporality in causal relationships mitigates some time effects (e.g., delayed distal effects of cause [33], [34], [35]), and temporal information has been shown to override contingency information and mislead, producing erroneous causal judgments [9]. Thus, temporal information and knowledge should take precedence over contingency in inferring causality. Although there are other key differences between associative and knowledge based models (such as state of association versus truth of inference [32]), of relevance at this point are the different mediating mechanisms for time and contingency effects.

Associative models, such as the Rescorla-Wagner model (RWM: [36]), explain both time and contingency effects through the interference of background context in the development of action-effect associations. For any one effect, there is a finite amount of associative strength available to the relevant action. Thus, although all stimuli present at the same time as the putative cause will develop associative links, they must compete with one another for their share of the overall associative strength available. Causal judgments change with variations in contingency because every time an effect occurs in the absence of the action (see Figure 1), the context-effect association becomes stronger and this, in turn, interferes with the development of the action-effect association by reducing the amount of associative strength available to it. Similarly, delays between action and outcome allow the experimental context to be more temporally contiguous with the outcome than the action, allowing the context rather than the action to develop associative strength [7]. A reduced rate of action-effect occurrence over time would produce the opposite effect [16]. Thus, while time and contingency are important to the development of associations and determining the strength of causal relationships, according to the associative model, the explanatory mechanism for both effects is through the development of interfering contextual associations.

Inference or knowledge based models can also explain the effects of time and contingency on causal judgments. However, these models hold that knowledge of time, in terms of contiguity and succession, is a starting point where causal knowledge is used to generate initial causal models or hypotheses about a given situation, with contingency information used secondarily to test causal hypotheses [37], [38] or assess the strength of the causal relationship [11], [12], [13]. Importantly, the assumption of the existence of causal knowledge can, quite naturally, explain data which associative models find difficult to incorporate. For example, although delay effects are generally robust, temporal knowledge provided in the form or instruction or cover story has been shown to mitigate their effect. Thus plausible delays consistent with a cover story, such as a grenade being fired towards a target and producing an explosion several miles away, are less deleterious to causal learning than unexplained delays [33], [34], [35], [39]. Although the effect of the plausibility of delay is often taken as supporting knowledge based models of causal judgments, it has also been argued that associative models, which code for time [40], can account for knowledge based time effects [41].

This short discussion shows that the functions of time, context and contingency distinguish theoretical accounts of causal learning. Associative models are time sensitive but explain contingency and time effects on the causal learning process through the development of contextual associations. Knowledge about temporality in causal relationships, on the other hand, is critical to establishing the existence of cause according to some knowledge-based accounts of causal learning. Contingency information is used subsequently to establish the strength of such causal relationships, though time can impact experienced contingencies also through changes in event-outcome conjunction categorization (i.e. cell A might be experienced as cell B [29]). However, in terms of which perspective, if any, is best supported by empirical evidence, and as we have argued above, time and context are inter-twined. Moreover, thus far, the interplay between time and context in causal learning has not explicitly been studied. In the next section, we argue that introducing an individual difference variable into experimental work, namely depression, may prove fruitful in terms of elucidating the mechanisms responsible for time effects on causal learning.

### Depression and causal learning

Depression effects on causal learning are particularly important because existing evidence suggests that they are moderated by experiences of time and context. For example, a growing body of evidence shows that even quite mild levels of depression affect people's ratings of the causal consequences of their actions [18], [19], [42], [43]. In order to explore the mechanisms underlying these effects, several studies have manipulated exposure to context by varying the length of time - the duration of the inter-trial interval (ITI) – during the causal learning procedure when no other events take place [16], [44]. For participants categorized as non-depressed, the trend was for long ITIs to increase the perception of causality in the presence of a zero or positive contingency, but weaken that impression in the presence of a negative contingency. In other words, these time effects were asymmetrical over contingencies and were consistent with the idea that the temporal manipulations affected the strength of context-outcome associations.

On the other hand, participants categorized as mildly depressed displayed quite a different pattern of effects. A contrast between medium (3s) and long ITI (15s) exposure did not affect ratings of a zero contingency [16] but the difference between very short (0.5s) and long (15s) ITI exposure decreased the perception of causality with zero and positive contingencies [44], and increased the perception of negative cause when the contingency was negative (E3). Although these latter findings were reported as of borderline significance using a conservative rejection criterion, the trend was again asymmetrical but diametrically in opposition to those effects displayed by non-depressed groups. A more conservative position would of course be that the time/context manipulations had no effect on the causal judgments made by mildly depressed participants.

Irrespective of the theoretical interpretation of these particular findings, it is evident that simultaneously manipulating time and exposure to the context affected the causal judgments of mildly depressed and non-depressed people differently. This could equally be due to mood related changes in time perception [20], [45] or processing of context [23]. However, exploring the underlying reason for this difference, whether located at the level of time or context processing, can inform about the relative contributions of time and context as causal cues.

In the series of experiments reported here, then, we planned to test predictions about how a range of temporal manipulations will affect causal judgments, in particular durations of action-outcome delays and inter-trial intervals. Predictions can then be extended to the effects of depressed mood on causal judgments. For example, numerous studies suggest that time perception is slowed in depression [22], [46], even mildly dysphoric states [20]. If slowed time perception underlies the effect of depression on causal learning, this would suggest that time effects will be magnified in participants who are categorized as depressed. However, it is equally possible that impaired processing and maintenance of context representations [23] are responsible for depression effects, in this is the case, then all time/context exposure manipulations will have reduced effects in depressed participants in comparison to controls. Studying how depression levels moderate the effects of the time and context manipulations, we have just described, will inform about the combinatorial process underlying causal judgments as well as elucidate the mechanisms through which depression affects causal learning.

## Experiment 1

Previous studies involving depression, temporal manipulations or context in causal learning [16], [18] have used a limited range of conditions, mainly those in which the contingency between cause and effect was zero and outcomes were frequent. This means that extant data on depression effects in causal learning currently provide an insufficient baseline against which to explore the effects of temporal and contextual manipulations in the subsequent experiments we plan to report here. Therefore, in Experiment 1, we used an instrumental causal learning task to test a range of preventative (*ΔP* = −.5), generative (*ΔP* = +.5) and non-contingency conditions (*ΔP* = 0), with different levels of outcome density (low, high). The cover story and visual stimuli used in the task included an explicit and realistic context. The goal of this was to directly consider the role of context in causal learning alongside the other causal cues. Thus, although indirect evidence for the role of context can be obtained from temporal manipulations as will be tested in subsequent experiments, direct evidence can also be derived from explicit ratings of the causal relationship between the context and the outcome. This approach to direct measurement of context is supported by data from previous studies we have carried out in which causal relationships were embedded in realistic virtual contexts, with participants then required to rate the causal relationships between the context, action and outcome [47]. As expected, context ratings were higher than action ratings with zero contingencies and this pattern was reversed with positive contingencies [47] – see supplementary data. Moreover, ratings were sensitive to relatively small elevations in depressed or dysphoric mood. Those previous findings indicate the suitability of a virtual context procedure, like that used in Experiment 1, to provide a more comprehensive data set than those currently available, against which to consider the findings of the subsequent experiments reported here.

### Method

#### Ethics statement

Ethics approval was obtained from the ethical review committees of the Universities of Limerick and Oxford for all experiments reported here, and written informed consent was obtained from all participants prior to participation.

#### Participants

University students were recruited via a mass screening method, which required all volunteers to complete the Beck Depression Inventory, hereafter BDI [48], as a measure of their current mood state before being invited to participate. BDI scores were taken again during participation and used to assign 50 participants to the high BDI group (scores of 9 or above: *n* = 24 with *n* = 4 males) or the low BDI group (scores of 8 or below: *n* = 26 with *n* = 17 males). These criteria are consistent with no dysphoria in the low BDI group and mild dysphoria in the high BDI group and have been used in many previous studies [16], [18].

As the procedure was not fully randomised, groups were matched on potential confounds such as age, working memory capacity, and estimated pre-morbid IQ, which could have contributed to any between groups effects (see Table 1). Working memory capacity was measured using the forward version of the digit span test [49], and premorbid IQ was estimated using demographic data (for method and equations see [50]). Independent groups t-tests showed that there were no between group differences in age, digit span score or estimated IQ. As expected, the high BDI group produced significantly higher scores on the BDI at both at screening and during their visit to the lab (see Table 1).

**Table 1:** Demographic characteristics of participants in Experiment 1 compared across low and high BDI groups.

Demographics	BDI group				Independent groups ttest	
	Low BDI		High BDI			
	(n = 26)		(n = 24)			
	*M*	*SE*	*M*	*SE*	*t*	*p*
Age	20.73	0.36	20.83	0.63	0.14	0.886
Digit Span	8.19	0.27	8.29	0.27	0.26	0.795
Estimated IQ	110.23	0.90	109.42	1.23	0.54	0.595
BDI_Screen_	5.46	1.00	21.75	2.66	5.74*	<.001
BDI_Lab_	4.04	0.57	23.5	2.59	7.34*	<.001

* Equal variances not assumed.

**Design and Materials.** In this experiment, we used a 2×(3×2×2) fully factorial mixed design. The within subjects factors were contingency (negative, zero, positive) outcome density [OD] (low, high) and cue (action, context). The between subjects variable was BDI group (low, high). Thus, each participant was exposed to six different contingency conditions, where the programmed *ΔP* values were: −0.5 low OD (0.0|0.5), −0.5 high OD (.5|1.0), 0 low OD (.25|.25), 0 high OD (.75|.75), +0.5 low OD (0.5|0.0), and +0.5 high OD (1.0|0.5), where the first value in each parentheses is *p*(Outcome|Action) and the second value is *p*(Outcome|NoAction). The cue variable refers to the two different causal ratings that participants were required to provide for each condition, action and context.

Each condition was located within a distinct virtual context represented by pictures on the computer screen. The action was a key press on the computer keyboard and the outcome was a 2s auditory stimulus. Following each condition, participants were asked to rate their own control (action), and that of the context, over an auditory outcome using a judgement scale which varied from −100 (labelled totally prevent) through 0 (labelled no influence) to +100 (labelled totally control). Order of presentation was counterbalanced using a Latin squares design. Presentation of experimental events was programmed using a Macintosh computer and REALbasic (2009, Release 2.1) software.

**Procedure.** During their visit to the laboratory, participants were briefed verbally about the nature of the experiment and then given a written information sheet to read. After giving written informed consent, participants provided demographic information, completed the digit span task, and questionnaires measuring mood state. Following this, instructions about the causal learning task requirements were displayed on the computer screen. The cover story required participants to imagine that they were in a house in which there was a hidden stereo system. They could control the music switching on in each of the rooms in the house (distinct contexts) using a remote control. However, participants were told that the remote control had been working intermittently, and that sometimes music switches on when no one is touching the remote control. The task was therefore to test the remote control in each of the rooms separately.

For each test, participants were told that they would be taken to the particular room and that they should wait to receive a message on the computer screen saying that they were allowed to test the remote control by pressing the spacebar on the computer keyboard. This would happen on many occasions (experimental trials) while they were in the room, and participants could choose to press the space bar at that point, or simply observe. In order for participants to properly gauge what happens when they did not press the button, they were asked to press on approximately half of the possible occasions.

Each experimental trial was constructed such that the message signalling the possibility of the action would stay on the screen for 3s. If the spacebar were pressed during this period, the button on the remote control shown on the screen would show as depressed. No further responses were possible during that particular response time window. At the end of the time window, the music would either play for 2s at a probability of *p*(Outcome|Action) or the room would remain silent. If the spacebar were not pressed during the time window, then the music would switch on for 2 s at a probability of *p*(Outcome|NoAction). This 5s procedure constituted one experimental trial, of which they were 40 in each condition, separated by a 3s inter-trial intervals (ITIs) during which the same visual stimuli (the virtual context) remained the same as during the trial.

At the end of each set of 40 trials, a judgement window was displayed and participants were required to rate the causal relationship between their own action and the outcome, and between the distinct context and the outcome using sliders displayed on the computer screen. The judgement sliders were constructed with increments of +/−1, so that the full range of the judgement scale (−100 to +100) could be used. After completing all six conditions, participants were thanked, debriefed and paid a nominal fee for their participation. All participants were also provided support information relevant to mood states.

### Results and Discussion

**Figure 2:**
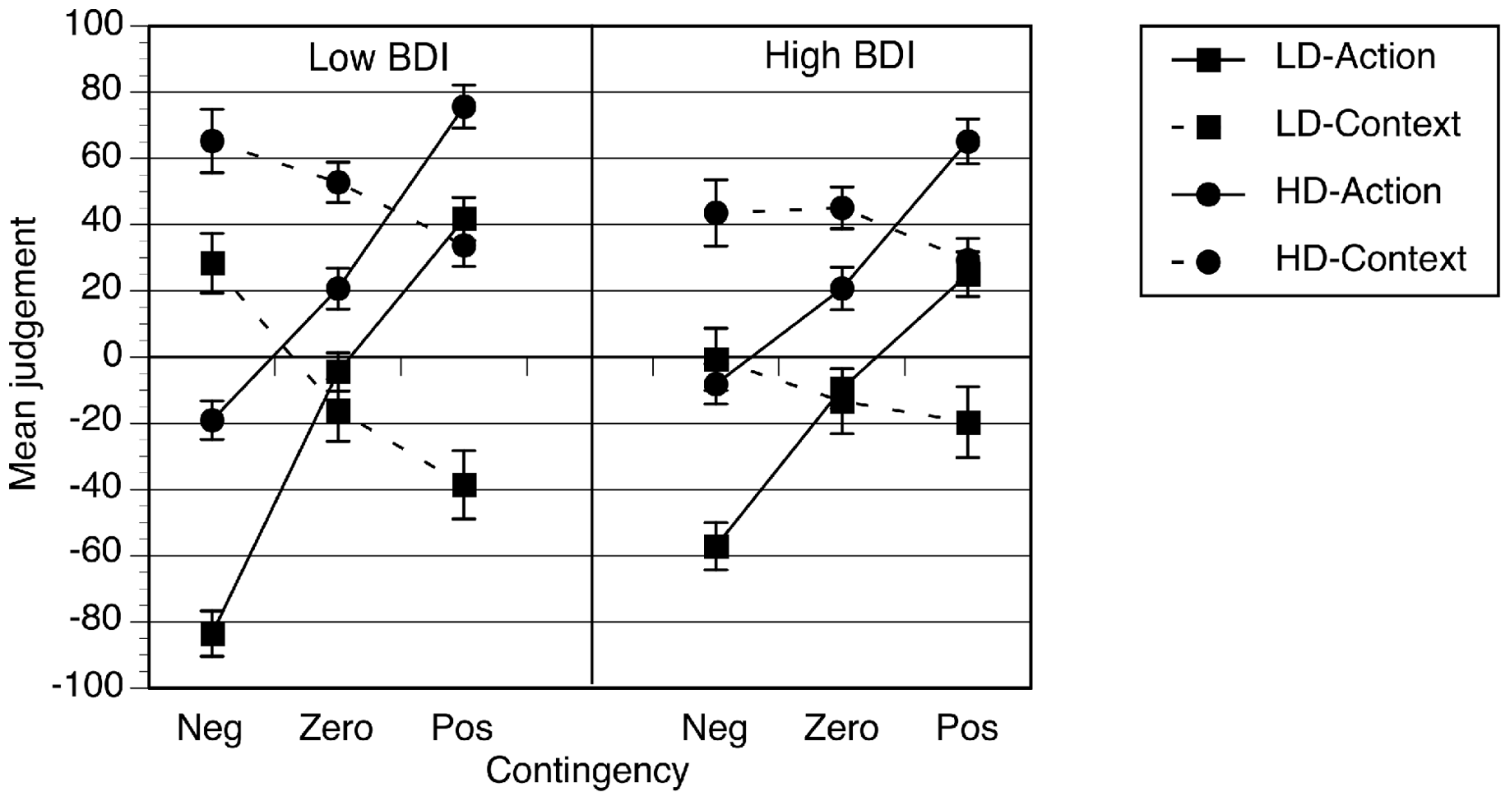
Ratings of the causal strength of the action and the context. Error bars correspond to the standard error of the mean. NB: LD  =  low outcome density, HD  =  high outcome density, Neg  =  negative contingency, Zero  =  zero contingency, Pos  =  positive contingency.

Participants rated the control of their actions over the outcome as well as that of the context in six different conditions, including negative, positive and zero contingencies with a low and high density of outcomes. These data are shown in Figure 2 and suggest that participants' ratings distinguished between action and context, and between the contingencies and the density of outcome occurrence. However, the experimental manipulations seemed to have a weaker effect on ratings made by the high BDI group in comparison to the low BDI group.

These observations were examined using a mixed (3×2×2) ×2 factorial analysis of variance, with contingency, outcome density and cue as within subjects factors and BDI group as the between subjects variable. An alpha level of .05 was used here and throughout unless stated otherwise. As we might have expected, contingency affected all ratings, *F*(2, 96)  = 43.07, *p*<.001, η^2^ = .47, *MSE*  = 1077.63, but the direction of the contingency effect depended on which cue, action or context, was rated, *F*(2, 96)  = 113.32, *p*<.001, η^2^ = .70, *MSE*  = 1084.63, as well as participant group, because the three-way interaction between contingency, cue and BDI group was significant, *F*(2, 96) = 7.59, *p* = .001, η^2^ = .14, *MSE*  =  1084.63. Before exploring that interaction further, it is important to note that the density of outcomes affected ratings, *F*(2, 96)  = 131.67, *p*<.001, η^2^ = .73, *MSE* = 2592.03, but that this effect depended on the cue rated, *F*(1, 48)  = 4.40, *p* = .041, η^2^ = .08, *MSE*  = 1774.47, and the contingency, *F*(2, 96) = 8.80, *p*<.001, η^2^  = .16, *MSE*  = 1051.46, but not BDI group, *p* = .12. In general, although the ratings of low and high outcome density conditions were located in different regions of the judgment scale the pattern of difference was similar. High outcome density conditions always received action ratings and context ratings that were more towards the positive end of the judgment scale than low outcome density conditions. For negative contingency conditions, this meant that high outcome density action ratings were weak and located nearer to zero on the judgment scale than low outcome density action ratings.

Further analyses of the contingency, cue and BDI group interaction revealed quite straightforward effects. For action ratings, the contingency by BDI group interaction was significant, *F*(2, 96)  = 6.68, *p* = .002, η^2^  = .12, *MSE*  =  1004.28. Both low and high BDI groups were sensitive to the difference between contingency conditions, where negative < zero < positive ratings (*p*<.001 for both groups, with all pairwise comparisons *p*<.001). However, as revealed by the significant interaction, the size of the contingency effect was greater for the low BDI group (η^2^ = .89) than the high BDI group (η^2^  = .72). For the context ratings, again the simple interaction between contingency and BDI group was reliable, *F*(2, 96) = 3.73, *p* = .028, η^2^ = .07, *MSE*  = 1877.98. For the low BDI group, context ratings were significantly affected by contingency, *F*(2, 50) = 23.65, *p*<.001, η^2^ = .49, *MSE*  = 1343.89, with the ordering of mean context ratings being in the opposite direction to action ratings, negative > zero > positive, with all pairwise comparisons significant with *p*<.005. In the high BDI group, however, contingency had no effect on context ratings, *F*(2, 46)  = 1.34, *p* = .26, thus negative  =  zero  =  positive context ratings.

In summary, when participants, categorized as having low or high BDI scores, were exposed to a series of six different contingency conditions, low BDI participants' ratings were consistent with a greater degree of discrimination between the contingencies than high BDI participants, and this was consistent irrespective of the density of outcomes. Previous work has only found differences between mood groups in specific high outcome density conditions when each participant was only exposed to one contingency condition [16], [18]; the findings of this experiment show more widely spread mood effects which are present in conditions more similar to the real world in which there are numerous contingencies to judge and compare. In addition, these results provide direct evidence for the first time of mood effects on people's ratings of the context's causal relationship with the outcome in a wide range of conditions. The high BDI group's context ratings did not vary as a function of contingency as the low BDI group's ratings did.

The findings do show very clearly how, for low BDI groups, their ratings of the causal role of the action varied systematically and with their causal ratings of the context. This is certainly consistent with the view of context as playing an important role in causal learning. The high BDI group, on the other hand, did discriminate between contingencies in terms of their action ratings but not their context ratings. High BDI context ratings did not change significantly across contingency although they did reflect levels of outcome density. This pattern is suggestive of causal judgments that interact less with context, or background, as possible causes of effects, though are highly sensitive to the background rate of outcomes.

The findings of Experiment 1 therefore provide us with baseline measures of causal relationships between action, context and outcomes across multiple contingency conditions. They are informative in their own right, in particular as previous studies have always been suggestive that mood effects on causal judgments only occur in very specific conditions, with zero contingencies [51] especially with long temporal intervals between trials. In fact, one criticism of this area of research has been that these findings are so specific as be rather meaningless in the real world [52]. However, the results of Experiment 1 do show that pattern of differences in causal learning attributable to depressed mood are more pervasive than previously thought.

## Experiment 2

In the next set of experiments, we varied the durations of the inter-trial interval (ITI) and the action-outcome delay in conditions with a moderately positive contingency and a high density of outcomes (*ΔP*  = 0.5). Both are manipulations of time but are simultaneously manipulations of exposure to context and both theoretical models made specific predictions about the effects of these manipulations.

From the perspective of associative learning theory, predictions are straightforward. Long action outcome delays mean that the constantly present context enjoys greater contiguity with the outcome that the action and this will strengthen context-outcome associations. Longer durations in between experimental trials (ITIs) would have the opposing effect, creating long periods of context exposure in the absence of the outcome, thus weakening the association between context and outcome. However, these consequences of the strength of the context association will be dependent on the specific contingency condition tested. When contingencies are positive or zero, strong and weak context associations will weaken and strengthen ratings of the action's causal relationship respectively. In the case of a negative contingency, however, the effects would be reversed such that the stronger context association would actually promote a stronger preventative causal relationship between action and outcome. In other words, associative theory would predict asymmetrical time/context effects that are contingency dependent.

These predictions diverge from those made by knowledge-based models and the following discussion explains the reasons for this and then describes specific predictions. We particularly refer here to causal structure models [12] as examples of what Lagnado and Sloman [9] refer to as hypothesis driven accounts of learning. According to this view, knowledge of temporality in causal relationships is key to determining whether or not a causal structure exists. This is only one part of the process. Following that, contingency data is used to determine the strength of the causal relationship. In the experiments reported here, **both** components of the process are required as participants are asked to rate how much, if any, control they have over the music switching on. Accordingly, in order to make the rating, they must use their knowledge of the plausibility, ontology and form of causal relationships to establish whether it exists or not, before establishing its strength [12].

According to Griffiths and Tenenbaum, it seems likely that people make different assumptions about generative and preventative relationships (with different strength parameterization calculations following on from this). However, given the plausibility of a generative relationship in the experimental scenario used here (remote button usually causes music to switch on), and the relative implausibility of a preventative relationship (remote button doesn't usually prevent music from switching on), it seems likely that people would assume a generative relationship and use their temporal knowledge in this way, such that delay is incompatible with causality. Specifically then, in this generative scenario, delay effects should be contingency independent, should be symmetrical across positive and negative contingencies, and should eliminate the perception of causality. This would be the case unless the notion of prevention is very clearly part of the causal scenario [53], which was not the case here (i.e. the rating scale allowed for prevention, but the scenario did not include it). We will return to these points later in the general discussion.

While predictions about delay do seem to distinguish the models, predictions around ITI duration do not. Instead they relate to how rates and probability of event occurrence are linked to knowledge in the first place and then use of contingency information to qualify initial assessments. As with associative theory, asymmetrical causal ratings would be predicted. Increasing the duration of the ITI will reduce the base rate of the effect [*p*(Outcome|No Action)] because of the conceptual similarity between ITIs and the D cell of the contingency table, and would increase the perception of generative causality and decrease the perception of preventative causality, as predicted by associative theory as well. However, there is some ambiguity to these ITI predictions. If rate and probability are processed online over time, rather than over N trials [5] (N trials would likely be controlled by the experimenter), then for a given time window, longer ITIs might also decrease the perceived rate of action-effect co-occurrences as well. In other words, both relevant conditional probabilities [*p*(Outcome|no Action) and *p*(Outcome|Action)] would decrease, maintaining the overall contingency, having no effect on causal strength. Thus, as causal strength is based on contingency information experienced over time, knowledge based theory could also predict the ITIs would have no effect on the strength of causal ratings.

One methodological issue, however, is that manipulating durations, which occur within or between trials, also affects the overall duration of exposure to a particular contingency condition [44]. Conditions with longer ITIs and delays necessarily involve a longer procedure time than shorter ITIs and delays if numbers of trials are held constant across conditions. We therefore carried out two versions of the experiments reported next; one in which the number of experimental trials was held constant while procedure time was varied (version A), and another in which the number of experimental trials was varied while procedure time was held constant (version B). We only report the details of the individual experiments where relevant, as there were no significant differences between the two.

### Method

#### Participants

All participants completed the Beck Depression Inventory on two occasions, a maximum of 14 days before participation and then again during the visit to the lab. Participants were assigned to mood groups on the basis of the BDI scores taken in the lab (Experiment 2a: *N* = 50; Experiment 2b: *N* = 53). In this experiment, we used median BDI scores to assign participants to the low and high BDI groups. Consequently, those who scored 5 or below on the BDI were assigned to the low BDI group while those who scored 6 or above were assigned to the high BDI group. Mood effects have been observed using the same criteria in previous experiments [47]. The data for five participants were excluded, one female participant due to computer malfunction, and four other participants due to low response rates *p*(R) <.13). The characteristics of the final sample are shown in Table 2 and comprised *N* = 46 participants in Experiment 2a and *N* = 52 participants in Experiment 2b.

**Table 2:** Experiment 2a and Experiment 2b demographic characteristics, comparisons across experiment and mood group.

Demographics	E2a				E2b				Exp comp	Mood comp
	BDI group									
	Low (n = 19)		High (n = 27)		Low (n = 26)		High (n = 26)			
	*M*	*SE*	*M*	*SE*	*M*	*SE*	*M*	*SE*	*p*	*p*
Age	22.47	1.62	22.11	.81	21.12	1.12	20.85	.50	.20	.84
Digit Span	8.37	.21	7.93	.36	7.77	.25	7.35	.29	.07	.21
Education	16.05	.40	16.26	.31	15.42	.25	15.77	.34	.08	.31
BDI Time1	3.11	.67	11.85	.89	3.00	.47	14.81	1.38	.64	<.001
BDI Time2	2.11	.38	11.26	.91	2.15	.34	13.38	1.43	.84	<.001

Note: Exp comp  =  comparison of demographics across experiments; Mood comp  =  comparison of demographics across mood groups.

The low and high BDI groups were compared on a range of relevant demographic variables using multivariate analysis of variance, including age, years of education, and scores on the digit span test. There were no significant differences between BDI groups on these variables, or across Experiments 2a and 2b (see Table 2). All participants received a nominal payment for their participation.

#### Design and Materials

This experiment used a 2× (2×2×2) mixed factorial design, where BDI group (low, high) was the between subjects variable. The within subjects variables were delay (short 0 s, long 4 s), ITI length (short 3 s, long 15 s) and cue (action, context). These durations of delay and ITI have been used in previously published studies [7], [16]. The factorial combination of experimental manipulations resulted in four conditions experienced by all participants, with two ratings – action and context – made for each condition. Order of presentation was counterbalanced using a Latin squares design.

The procedural details were the same as the previous experiment except that durations were varied. Table 3 shows the duration of each component of the procedure. In all conditions, there was a 3s period in which participants could choose to make the action. One difference between this experiment and Experiment 1, is that if an action occurred during the 3 s response window, then the outcome followed immediately or after 4s at a probability of *p*(Outcome|Action) rather than at the end of the response window. If no action was recorded by the end of the response window, then an outcome followed immediately or after 4s at a probability of *p*(Outcome|No Action). Outcomes lasted for 2 s. In Experiment 2a, each condition included 60 trials with the duration of each condition varying accordingly, whereas in Experiment 2 b, the number of trials was varied in order to hold the duration of each condition constant (see Table 3). In all cases, the overall procedure time, including all four conditions, lasted for 64 minutes.

**Table 3:** Component durations (s) and numbers of trials in each condition in Experiment 2.

ExperimentalConditions		Programmed Durations				Experiment 2a		Experiment 2b	
Delay	ITI	Action *s*	Delay*s*	Outcome *s*	ITI*s*	Trials*N*	Total Time*s*	Trials*N*	Total time*s*
Short	Short	3	0	2	3	60	480	120	960
Short	Long	3	0	2	15	60	1200	48	960
Long	Short	3	4	2	3	60	720	80	960
Long	Long	3	4	2	15	60	1440	40	960

In all conditions, there was a moderately positive contingency (*ΔP* = .5) between the action and the outcome, such that actions always resulted in an outcome with a probability of 1.0 and trials with no action resulted in an outcome at a probability of .5.

#### Procedure

Procedural details are identical to Experiment 1.

### Results and Discussion

**Figure 3:**
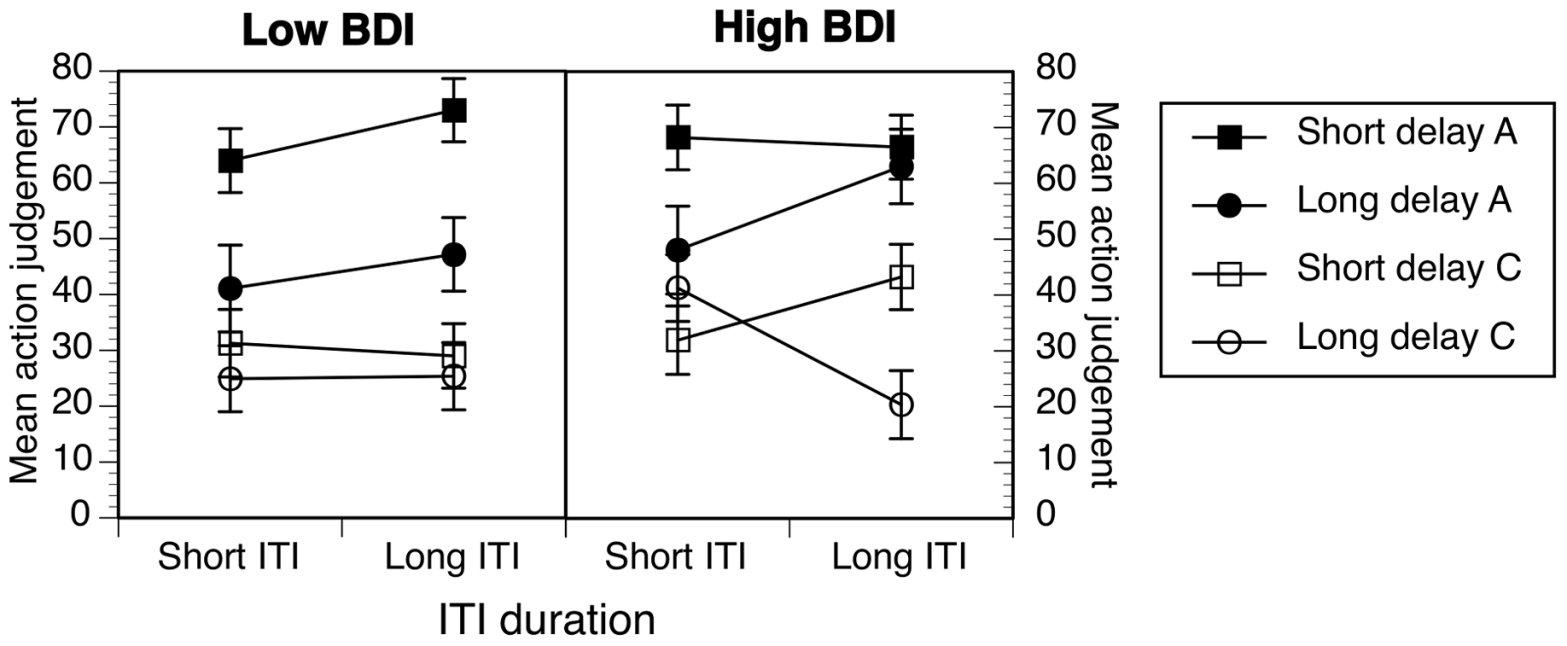
Effects of ITI length and delay duration on ratings of positive (100/50) contingencies. Data are combined across Experiments 2a and 2b. Error bars correspond to the standard error of the mean. A  =  action ratings, C  =  context ratings.

As with the previous experiment, participants rated the effectiveness of their own actions, and the effectiveness of the context, in controlling the occurrence of the music. These data, combined across Experiments 2a and 2b, are described below and shown in Figure 3.

In general, participants judged that the causal role of their own actions was stronger than the causal role of the context. However, the effects of delay and ITI length seemed to depend on both BDI group and the specific causal role rated. The data were analyzed using Analysis of Variance with the following variables, cue, delay and ITI, included as within subjects factors, with BDI group (low, high) and Experiment (2a versus 2b) entered as between subjects variables.

The analyses showed that action ratings were indeed significantly higher than context ratings, cue: *F*(1, 94)  = 50.35, *p* <.001, η^2^  = .35, *MSE*  = 3040.38, and that delay affected ratings also, *F*(1, 94)  = 19.40, *p*<.001, η^2^  = .17, *MSE*  =  1361.91. However, delay effects depended on cue, cue × delay: *F*(1, 94)  = 6.16, *p* = .015, η^2^  = .06, *MSE*  = 1372.49, as well as ITI length, cue × delay × ITI: *F*(1, 94) = 6.69, *p*  = .011, η^2^  = .07, *MSE*  = 782.27, and BDI group, cue × delay × ITI × BDI: *F*(1, 94)  = 9.92, *p* = .002, η^2^  = .10, *MSE*  = 782.27. None of the effects or interactions involving Experiment (2a versus 2b) were reliable or were explored further.

In order to explore the four-way interaction between cue, delay, ITI, and BDI group in more detail, we split the data by BDI group and carried out further analyses. For the low BDI group (Figure 3, left), the cue by delay interaction was reliable, *F*(1, 43)  = 7.97, *p* = .007, η^2^  = .16, *MSE*  = 970.62, although the cue by ITI interaction was not reliable, *p* = .19. Although we might have expected an effect of ITI length on action judgments, and there was some suggestion of this in Figure 3, the ITI effect on action judgments was not reliable, *p* = .18. Simple effects analysis showed that whereas long delays reduced action ratings significantly, *F*(1, 43)  = 16.14, *p*<.001, η^2^  = .27, context ratings remained the same, *p* = .30.

The pattern was different in the high BDI group. The cue, delay by ITI interaction was reliable, *F*(1, 51)  = 15.38, *p*<.001, η^2^  = .23, *MSE*  = 923.16. When delays were short (squares, Figure 3), ratings were not dissimilar to the low BDI group, with action rated significantly higher than context, *p* < .003, η^2^ >.16, and with no discernible effect of ITI length, *p* >.12, η^2^ <.05. However, when delays were long, action judgments increased and context judgments decreased significantly with longer ITIs, both *p*s < .02, both η^2^ > .10. In other words, ITI effects were strongly evident but only in long delay conditions. In long ITI conditions, there was no difference between short and long delay action judgments, *F*(1, 51)  = 1.04, *p* = .31.

Taken together these findings show that manipulations of the time and context affect causal judgments but that the nature of these effects depends on levels of depressed mood. For low BDI groups, there were trends, some significant, towards effects observed in previous studies. Longer action-outcome delays reliably decreased people's judgments of causal relationships, whereas longer ITIs did not reliably affect causal judgments. These effects were not mirrored in context judgments. Thus, this pattern of findings is most consistent with the notion of a time-based moderation of causal judgments in low BDI participants.

However, it could be argued for several reasons that the data from high BDI groups was consistent with effects related to time and context. This is because slowed time experience in depression should magnify the effects of time manipulations, whereas impaired representation or processing of context would reduce the strength of time effects. Consistent with the slowed time experience view, the delay and ITI effects were stronger in high BDI groups' judgments. However, this magnification of time effects concurrently affected context ratings in a manner consistent with contextual mediation of time effects.

The implications of this are as follows. In Experiment 1, we observed strong mirroring of action-context ratings in low BDI as a function of contingency effects. This might suggest then that the time manipulations, tested with identical contingency conditions in Experiment 2, were not strong enough to produce the action context mirroring in low BDIs that we observed in Experiment 1. However, for high BDIs, their slower time experience increased perceived duration to the extent that time effects were stronger and thus mirrored in context ratings. The fact that ITI effects, which involve much longer durations and therefore more accrued time, were stronger in long delay conditions is consistent with this interpretation of the findings.

## Experiment 3

Negative or preventative contingencies (see Figure 1) may be more informative than positive contingencies. This is because if the plausible causal relation is generative, knowledge based accounts predict that action outcome delays will reduce or eliminate the perception of action-outcome causality. However, associative theory would not make the same prediction. This is because contingency effects on causal learning are based on the strength of context as well as action associations. In the case of negative contingencies, context associations are very strong in a positive direction, whereas action associations are strong in an inhibitory direction. This means that context associations could be asymptotic, such that increasing the action outcome delay would likely not increase the strength of the context association. Moreover, any effect of delay on strengthening the context association would be likely to increase rather then decrease the perception of preventative cause. Long ITIs on the other hand would weaken the context association, and therefore reduce the inhibitory strength of the action association towards zero. Similarly, from the knowledge-based perspective, the effect of ITI on causal strength would be to reduce the perceived base rate of the effect or outcome [*p*(Outcome|No Action)], eliminating the perception of causal strength. If perceived rates of action-outcome occurrences over time are also reduced, this could mean that ITI has no effect on ratings.

### Method

#### Participants

Ninety-nine participants took part either in the fixed trials (Experiment 3a: *N* = 50) or the fixed time (Experiment 3b: *N* = 49) experiment. They completed the BDI as in the previous experiment and were assigned to the low and high BDI groups using the same median split criteria (BDI  = 5 cut off). However, data from seven participants were excluded for low response rates (*n* = 2: *p*(response) <.15) and high response rates (*n* = 4: *p*(response) > .85), and one further participant didn't use the keyboard as instructed which resulted in missing data. The characteristics of the final sample are shown in Table 4 and comprised *N* = 44 participants in Experiment 3a and *N* = 49 in Experiment 3b. There were no significant differences across experiments or mood groups on demographics, although high BDI groups scored significantly higher on the BDI at both time points than low BDI groups.

**Table 4:** Experiment 3a and Experiment 3b demographic characteristics, comparisons across experiment and mood group.

Demographics	E3a				E3b				Exp comp	Mood comp
	BDI group				BDI_num1low					
	Low (n = 23)		High (n = 21)		Low (n = 25)		High (n = 24)			
	*M*	*SE*	*M*	*SE*	*M*	*SE*	*M*	*SE*	*p*	*p*
Age	20.13	.28	20.81	.80	19.36	.80	20.33	.72	.37	.23
Digit Span	8.26	.27	7.86	.46	8.00	.24	8.33	.21	.75	.96
Education	15.61	.27	15.10	.30	15.78	.31	15.38	.22	.44	.10
BDI Time1	3.52	.76	10.62	1.83	3.56	.70	10.33	1.25	.98	<.001
BDI Time2	2.04	.31	11.38	1.16	2.20	.33	10.83	1.14	.95	<.001

Note: Exp comp  =  comparison of demographics across experiments; Mood comp  =  comparison of demographics across mood groups.

#### Design and procedure

The design was identical to Experiment 2, except that a negative contingency was tested (*ΔP*  = −.5). Actions resulted in an outcome at a probability of .5, whereas trials with no actions always ended in an outcome (*p* = 1). The instructions and judgment scale were identical to the previous experiment, as were the procedural details.

### Results and Discussion

**Figure 4:**
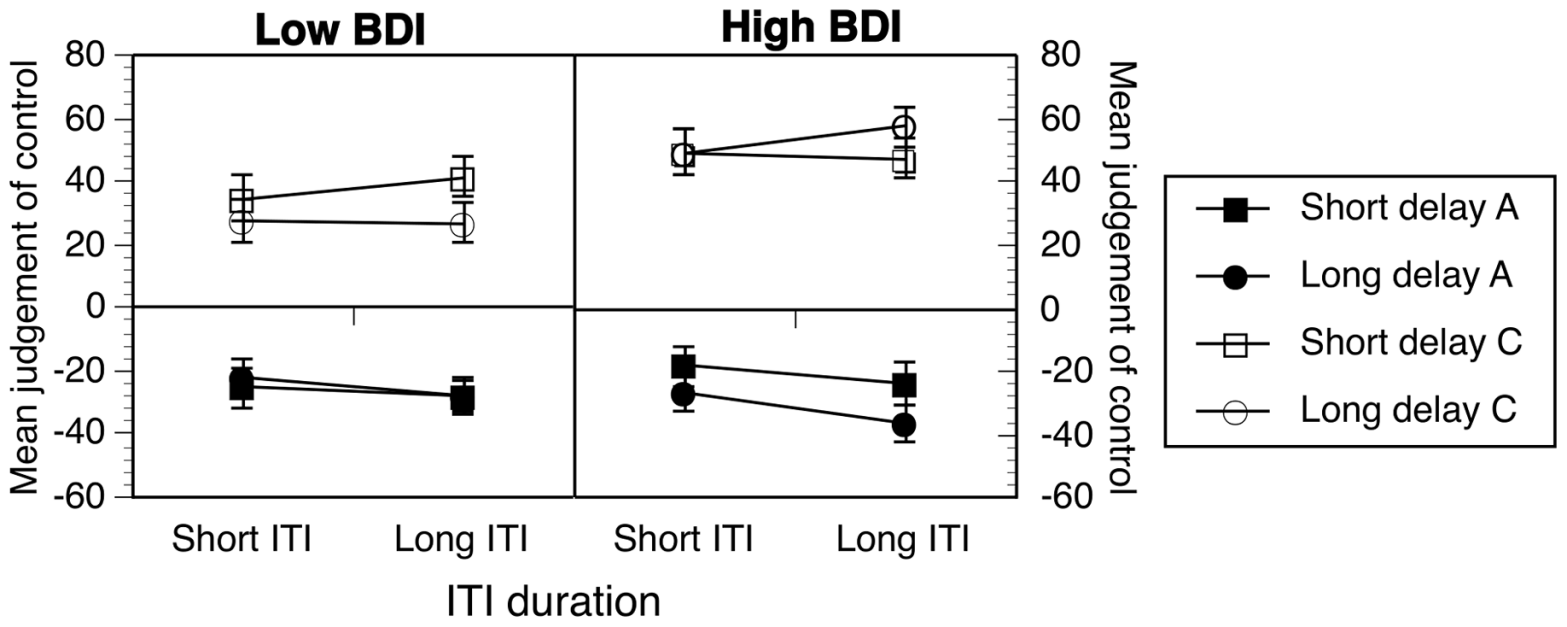
Effects of ITI length and delay duration on ratings of negative (50/100) contingencies. Data are combined across Experiments 3a and 3b. Error bars correspond to the standard error of the mean. A  =  action ratings, C  =  context ratings.

Participants were exposed to a moderately negative contingency and rated the extent to which their actions and the context controlled or prevented the occurrence of the music. These data are combined across Experiments 3a and 3b. Action ratings made by low and high BDI groups are shown in Figure 4 (filled symbols). For high BDI groups only, longer ITIs and longer delays appeared to be associated with more negative action ratings.

In order to explore these observations, the data were analyzed in the same way as in Experiment 2. Context ratings were significantly different to action ratings, such that context was rated as a facilitator of the outcome and the action was rated as a preventer of the outcome, Cue: *F*(1, 88)  = 189.72, *p*<.001, η^2^  =  .68, *MSE*  =  4526.87. Notably, action-outcome delay had no significant effect on ratings, *F*<1. Membership of the BDI group did significantly influence ratings, BDI: *F*(1, 88)  =  5.62, *p* = .02, η^2^ = .06, *MSE*  =  2721.05, and the difference between action and context ratings, BDI × Cue: *F*(1, 88)  = 4.09, *p* = .046, η^2^  = .044, *MSE*  = 4526.87. As there was a three way interaction between cue, delay and BDI group, *F*(1, 88)  = 4.46, *p* = .038, η^2^  = .048, *MSE*  = 1711.78, this was the starting point for further analysis involving the BDI variable. Finally, there was also a significant interaction between cue and ITI length, *F*(1, 88)  = 4.27, *p* = .042, η^2^  = .046, *MSE*  = 903.23, which also required further analysis.

Firstly, further examination of the cue by delay by BDI interaction, confirmed that both low and high BDI groups rated the context very differently to the action, all *F*s >79, all *p*<.001, all η^2^ >.63, and this difference was consistent across short and long delays, all *p*s >.13. Further tests showed that the source of the three-way interaction was that high BDI participants rated the context as a stronger cause of the outcome than low BDI participants, specifically in long delay conditions, *F*(1,88)  = 11.44, *p* = .001, η^2^  = .12, *MSE*  = 1448.83. This was not the case with short delays or with action ratings, all *F*s<.2.12, all *p*s>.14. We also checked whether long delays produced reliably more negative ratings in long versus short delay conditions, as suggested in Figure 4, however, that effect was not reliable, *F*(1,43)  = 2.10, *p* = .16, η^2^  = .05, *MSE*  = 1636.75.

Finally, we also examined the cue by ITI length interaction. Despite a trend towards ITI effects on causal ratings of the action for all participants, simple effects analysis showed that this was not significant, *F*(1, 88)  = 3.78, *p* = .055, η^2^  = .04, *MSE*  = 753.17. There was no suggestion of any ITI effect on context ratings, *p* = .25.

Overall, in Experiment 3, the context was rated as a facilitator of the outcome whereas the action was rated as a preventer. Delay and ITI length had no effect on ratings made by the low BDI group. However, the high BDI group rated the context as more strongly facilitative than the low BDI group, specifically in long delay conditions, although this effect was not mirrored reliably in action ratings where the delay effect did not reach criterion. Therefore, in this particular condition, in which the context was a strong cause of the effect by nature of the specific contingency tested, temporal manipulations had no effect on action ratings.

## Experiment 4

The previous experiment was designed to pit the predictions of associative and knowledge based models against each other. Moreover, we also wanted to test whether depression effects on causal learning were consistent with slowed time perception in depression strengthening the effects of temporal manipulations, with subsequent effects on causal learning through contextual associations. The lack of delay effects on action ratings of negative contingencies in the previous experiment is not entirely consistent with either theoretical approach, however it might be that the specific negative contingency tested in Experiment 3 was less likely than any other negative contingency to produce temporal effects.

The specific condition tested in Experiment 3 (see Figure 1, lower left) was programmed such that 50% of action trials and 100% of no action trials, during which the context was always present, resulted in an outcome. This configuration, with such a level of high outcome density, would mean that the context and outcome were frequently paired and the association between context and outcome would be very strong and possibly near to the limits of associative or causal strength. In addition, and from a more probabilistic perspective on delay, if a long delay between action and outcome means that the trial is processed as a context-outcome trial instead, cell C rather than cell A [29], the original 100% likelihood of outcome occurrence after a no action event is at ceiling and cannot therefore be increased. This does not explain why ITI effects were not observed in Experiment 3, however, but we cannot discount the fact that the findings might be related to the specific contingency tested.

Therefore, Experiment 4 will repeat the previous experiment (including fixed trials and fixed time versions) with a medium outcome density negative contingency. While the absolute level of contingency (DP  = −.5) will be the same, only 50% of trials will end in an outcome where 25% of action trials and 75% of no action trials will be followed an outcome. This means that the context will be paired with the outcome on fewer occasions than Experiment 3, and the problematic 100% outcome rate after no action trials is no longer the case.

### Method

#### Participants

Recruitment and BDI completion was carried out on the same basis as previous experiments. One hundred participants completed Experiment 4a (fixed trials: *N* = 50) or 4b (fixed time: *N* = 50). However, the data for nine participants were excluded due to response rates <.15 or >.85. The final sample comprised 44 participants in Experiment 4a and 47 in Experiment 4b (*N* = 91). The characteristics of the sample are shown in Table 5.

**Table 5:** Experiment 4a and Experiment 4b demographic characteristics, comparisons across experiment and mood group.

Demographics	E4a				E4b				Exp comp	Mood comp
	BDI group				BDI group					
	Low (n = 21)		High (n = 23)		Low (n = 29)		High (n = 18)			
	*M*	*SE*	*M*	*SE*	*M*	*SE*	*M*	*SE*	*p*	*p*
Age	19.67	1.04	18.39	.20	22.38	.77	20.72	.53	<.001	.02
Digit Span	7.43	.43	7.00	.24	8.21	.23	8.33	.40	.001	.37
Education	–	–	–	–	17.05	.43	14.67	1.16	–	–
BDI Time1	4.62	.66	13.96	1.64	3.14	.53	13.72	1.49	.13	<.001
BDI Time2	2.38	.39	13.87	1.72	2.72	.45	12.12	1.33	.15	<.001

Note: Exp comp  =  comparison of demographics across experiments; Mood comp  =  comparison of demographics across mood groups.

Education data was missing in Experiment 4a and therefore no experiment or mood group comparisons are reported for that variable. BDI scores were always significantly different between the low and high BDI groups but not across experiments. Experiment 4b participants were significantly older than Experiment 4a participants. Also high BDI participants were significantly younger than low BDI. Experiment was included as a variable in all analyses as in the previous experiments.

#### Design and procedure

All details were the same as previous experiments except that a moderate outcome density, negative contingency condition was tested. Outcomes occurred on 25% of action trials and 75% of no action trials.

### Results

**Figure 5:**
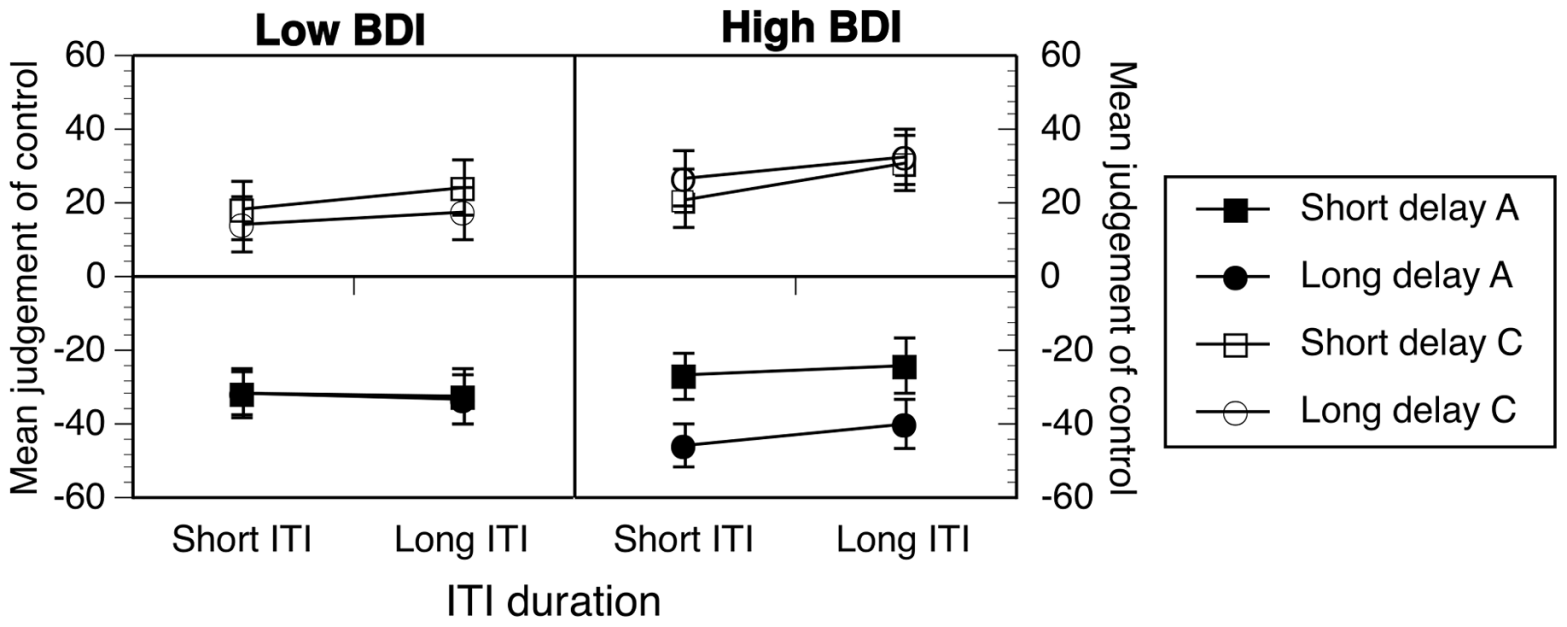
Effects of ITI length and delay duration on ratings of negative (25/75) contingencies. Data are combined across Experiments 4a and 4b. Error bars correspond to the standard error of the mean. A  =  action ratings, C  =  context ratings.

Participants rated the action and the context in relation to control over the outcome and the data are shown combined across fixed time and fixed trials versions in Figure 5. The data show a similar pattern to the previous experiment. Action ratings are in the negative portion of the scale and context ratings in the positive end, with little evidence of delay or ITI effects in low BDI ratings. High BDI ratings also looked similar to the previous experiment, except that the effect of longer delays, which pushed their actions ratings in a more negative direction, seemed more pronounced in Figure 5 than in the previous experiment.

The data were analysed as in Experiment 3. The difference between context and action ratings was significant for all participants, Cue: *F*(1, 87)  = 130.86, *p*<.001, η^2^ = .60, *MSE*  = 4393.04. In addition, the effect of delay was moderated both by cue and by BDI group, Delay × Cue × BDI: *F*(1, 87)  = 4.00, *p*  = .049, η^2^  = .604, *MSE*  = 4393.04. Examining the low and high BDI groups separately showed that for low BDI groups there was no effect of delay on either cue, *F*s <1, *p*s >.5. However, in the high BDI group, there was a cue dependent delay effect, *F*(1, 43)  = 4.56, *p* = .038, η^2^ = .10, *MSE*  = 1875.73, such that there was no delay effect on context ratings, *F*<1, but long delay action ratings were significantly more negative than short delay action ratings, *F*(1, 43)  = 8.73, *p* = .005, η^2^ = .17, *MSE*  = 1431.82.

This pattern of results is very similar to the previous experiment. Again, delay and ITI manipulations did not affect low BDI groups' context and action ratings. However, for the high BDI groups, stronger action ratings of preventative cause in long delay conditions, an effect that was weak and did not reach criterion in the previous experiment, was reliable and based on medium to large effects according to Cohen's [54] criteria. This is consistent with the delay effect increasing in strength in negative contingencies when there is a lower level of outcome density, high levels of which promote very strong context-outcome links. While this may be the case, in a supplementary analysis, we combined the data from both negative contingency experiments, and checked whether the cue × delay × BDI group interaction depended on the specific contingency (25/75 versus 50/100) condition tested. There was not the case and there were no reliable differences in the interaction between the two experiments, *F*(1, 179) <1, *p* = .99. This finding with high BDI groups only is consistent with slowed time experience magnifying time effects in both positive and negative contingencies in a manner that has a knock on effect on the strength of context associations

## General Discussion

In this series of experiments, we set out to explore the processes underlying causal learning, in particular how time and context manipulations affect causal judgments, with depression included as an important moderator variable. Unlike previous studies [7], [33], [34], and mindful that time manipulations simultaneously affect exposure to the context, we explicitly included measures of context causality. We also tested groups of low and high scorers on a depression scale for whom time and context processing impairments have been documented [20], [21], [23]. We found that for participants with little evidence of depression, effects of time manipulations were only apparent in specific positive contingency conditions. However, for participants scoring higher on the depression scale, time effects were generally stronger and present with positive and negative contingencies. We will discuss these findings in more detail below, also using the contrast between low and high depression scorers, and inconsistencies with previous findings, to inform the theoretical implications of this work.

In this series of experiments, we firstly verified that the strength of instrumental causal judgments, made across a range of contingency conditions, varied with similar ratings made about the relation between the context and outcome. This was the case for participants scoring low on the depression scale. However, high BDI groups' context ratings did not vary with action ratings in the same way across contingencies, suggesting a degree of ‘de-contextualization’ in their causal judgments in the absence of any specific temporal or contextual experimental manipulations. This effect was accompanied by action ratings that did not discriminate between contingencies to the same extent that low BDI groups' ratings did. This pattern of findings supports the idea that context typically does have a key function in causal learning, as suggested by associative theories [5], but that this mechanism is vulnerable to low levels of depression and perhaps other psychopathologies.

Further experiments were then designed to test the interrelations between time and context in positive (Experiment 2) and negative (Experiments 3–4) contingencies. Two different time periods were manipulated, the delay between action and outcome and the empty delay between experimental trials, that are thought to weaken and strengthen respectively people's assessments of the causal role of the action. These time effects distinguished low and high BDI scorers. We found that longer time delays did not always reduce the perception of cause for low BDI scorers. While delayed outcomes were perceived as less causal with positive contingencies, as in previous studies, they had no effect on causal judgments when the contingency was negative. It could be argued that this finding suggests that, all other factors being equal including knowledge of temporality and the plausibility of delay in that causal situation, time delays do not always eliminate the perception of a causal relationship as knowledge based theory might suggest [9]. We will return to this theoretical point shortly, however, it is the findings from high BDI scorers that are more suggestive of underlying mechanisms.

Firstly, it is useful to reconsider the original predictions that we made about time and depression. Depression has consistently been associated with slowed time perception [20], [22], which could **increase** the impact of time manipulations, but also impaired context processing [16], [23], which could **decrease** time effects if they are context based. In the time manipulation experiments reported here, time effects on ratings made by the high BDI groups were stronger than for low BDI participants and it is possible that slowed time perception is the cause of this effect. However, with positive contingencies, both delay and ITI influenced causal ratings, with action and context ratings being influenced in opposition. This might suggest then, that for these participants, slowed awareness of time exerts its effect on causal learning through extended exposure to context and the consequential effect of that on the strength of context associations, rather than the effect of time per se.

Even more informative is the finding that delayed outcomes increased rather than eliminated high BDI participants' perceptions of preventative cause in negative contingency conditions. This means that, for these participants, delay effects were asymmetrical around zero across the judgment scale (ratings of positive short > positive long > negative short > negative long). While knowledge based theory would predict symmetrical delay effects that eliminate the perception of causality (ratings of positive short > positive long > negative long > negative short), this was not the case for high BDI participants. The asymmetry of delay effects is however consistent with the idea that the stronger relation between context and outcome in long delay conditions would make an inhibitory association between action and outcome even stronger as predicted by associative theory.

These findings are consistent with some, but not all, previous work. For example, Vallee-Tourangeau, Murphy and Baker [8] reported findings consistent with our low BDI groups that variable (degraded) versus constant contiguity deleteriously affected positive action-outcome contingency ratings significantly but not negative contingency ratings. However, in contrast, Mutter, DeCaro and Plumlee [55] found symmetrical rather than asymmetrical delay effects with their younger participants. In their study, depression was not a variable of interest and they found that long delays reduced the perception of causality in negative as well as positive contingencies. Their older participants, like our low BDI groups and Vallee-Tourangeau et al. 's participants, displayed no delay effect on negative contingencies. For the most part then, it seems that the effects of delayed outcomes are specific to positive contingency conditions. However, when time effects are enhanced, here due to mild depressed mood, then the full range of delay effects are observable but the nature of the effects are contingency dependent and can enhance and eliminate the perception of causality.

Other inconsistencies between the findings reported here and previous studies relate to the effects of ITI duration on causal ratings. In the present series of experiments, ITI effects on causal ratings were, for the most part, absent or weak and not reliable. However, in our previous work [44], although we reported ITI effects to be weak with positive contingencies, they were strong when the contingency was negative. One reason for this inconsistency in ITI effects, as well as the delay effects mentioned above, might be theoretically important procedural differences. In the present study, and Vallee-Tourangeau et al. [8], in which patterns of delay effects similar to ours were reported, the time manipulations were tested on a within subjects basis. Mutter's study [55] and our own previous work, in which different patterns of delay and ITI effects were reported than those described here, involved between subjects tests of time variables. The within versus between subjects distinction of time effect tests is important as it might imply that time effects are cumulative, such that with multiple conditions time effects are influenced by preceding conditions, thereby explaining the difference in findings.

So far, we have discussed several specific pieces of evidence that inform the theoretical implications of this work and we address this now in detail. Causal structure models, as one example of a knowledge based approach, postulate that time is the primary cue to causality and that contingency is information that is considered subsequently in the process. So, for example, Lagnado [9] found that when time information misleads, erroneous causal attributions result. However, it is also clear that knowledge about the plausibility of temporality in a given situation [33] and assumptions about the functional form of generative (positive) and preventative (negative) causes [12] will mean that the effects of time information will be situational. So, for example, Griffiths and Tenenbaum [12] showed how the effects of outcome density manipulations on causal judgments of zero contingencies were reversed by framing the same situations as involving generative or preventative causes. In experiments, but not real life, such information is provided either explicitly or implicitly by the causal scenario. In the present set of experiments, the causal scenario was the same throughout. It could also be argued that a generative causal relationship was most likely assumed because people's causal knowledge of remote controls and music switching on would be consistent with that. If that were the case, outcomes occurring 4s after the action would not be consistent with existing knowledge, and delayed outcomes should therefore eliminate or ameliorate the perception of causality before contingency itself enters the causal process. On the contrary, participants in the present study identified the preventative causal relationship evident in negative contingency condition whatever the delay between action and outcome. Thus, we argue that findings that delay effects (low BDIs) are dependent on the contingency tested and that sometimes delays enhance causal perception (High BDIs, Experiment 4) are not entirely compatible with causal model theory.

There is an alternative argument, of course, which is consistent with other evidence that temporal contiguity between action and outcome is not essential for accurate causal learning. As mentioned above, if the experimental scenario includes a plausible reason for a delay between action and outcome to occur [33], or if another stimulus is inserted into the delay period [56] then delay effects can be reduced or eliminated in positive contingencies. It may be then that a negative contingency can itself act as a plausible reason for the delay. In other words, people may assume the plausibility of a preventative relationship between action and outcome, and then knowledge of the temporal structure of preventative causality is relevant. Thus action delayed outcome trials might be perceived as consistent with preventative cause. This initial ‘modal decision’ that a preventative relational structure exists would then allow contingency information to enter the causal process.

However, the **experienced contingency** would then depend on the duration of the temporal window used to determine whether two events co-occur or not [29], [57]. Thus depending on how event-outcome conjunctions were reclassified in the delayed time frame, a negative contingency could be experienced as more negative and thus this would be consistent with our results; or it might be experienced as random occurrences of outcomes that are simply not linked to any action response window resulting in an experienced zero contingency. The latter outcome would be consistent with Mutter's results showing that delays eliminated the perception of negative cause [55]. Notwithstanding, this is currently an area of theoretical imprecision as temporal windows are not only argued to be dynamic and changing in response to incoming information [57] but likely depend on the continuous or discrete trial nature of the procedure used [58]. Furthermore, it is also unclear how and under what conditions the modal switch involved in preventative and generative cause functions. It therefore could be argued that our findings are consistent with causal structure models.

Another important question, however, is whether such a conclusion would be consistent with the nature of the enhanced time effects we observed on the causal ratings of high BDI participants. Our findings support the hypothesis that slowed time perception in depression would augment the effect of increased delay or ITI. It could be argued then that the high BDI evidence points towards time dominating the causal process in these experiments in which contingency and existing causal knowledge were held constant. Despite this, for several reasons, we would argue for context as the explanatory mechanism for the effects. At baseline, high BDIs produced causal ratings that were less contextualized than other participants. However, delay and ITI effects in positive contingency conditions affected both action and context ratings in opposition, implicating a time effect through context. In these conditions, high BDI judgments were more contextualized than they had been at baseline. Then when negative contingencies were tested, delayed outcomes increased the perception of preventative cause. Taken together, these effects could be parsimoniously linked to time based fluctuations in context associations as predicted by associative theory. We might also speculate that cumulative effects of time over conditions, in relation to the difference in findings from between versus within subjects' designs, fit more readily with a context based associative learning framework than a causal model perspective.

Thus far we have discussed the findings from low and high BDI participants based on the assumption that both sets of people arrive at their causal ratings using the same causal processes but that these same processes are enhanced or impaired due to state changes in basic cognitive processing. However, we must acknowledge an alternative possibility that the two sets of participants used different processes or were at different stages of the same processes when they made their causal judgments. For example, Balleine and Dickinson [59] argued that instrumental action is underpinned by two different processes, goal directed action-outcome learning and more habit based stimulus response learning. Anatomically distinct from each other, goal directed action is evident early on in the process and this then transfers to more habitual behavior as learning progresses, which is stimulus driven and more independent of the outcome. Evidence from humans and animals also shows that higher levels of stress promote habit based performance over goal directed action [60], indicating that state changes can influence the function of these processes. Along similar lines, Sternberg and McClelland [31] argue that when there is time, and presumably cognitive resources, available to them, people will make inference based causal judgments. However, with less time and cognitive resources, associative processes would be used. It is possible then that low and high BDI groups' causal ratings represent either different causal learning processes due to the availability of cognitive resources, or different stages of the learning process.

Two process theories do provide an intuitive account of the data reported here. However, one question relevant to these data remains outstanding. Previous research has suggested that under certain conditions, people with higher levels of depression are more accurate or realistic in their causal judgments than others [18]. Realistic causal judgments, observed in some studies, must be reconciled to judgments which are also strongly affected by time and context, possibly due to slowed time perception, as in the present study. One putative reason for this is that slowed time awareness confers a normative advantage in relation to single judgments of a contingency, as these judgments would be less contextualized and more consistent with *ΔP*. However, in studies with repeated judgments, as in the current work, the effects of slowed time perception would accumulate over the course of repeated judgments, with appropriate contextualization of the individual judgments being compromised and perhaps unpredictable. Given that multi-judgment experiments bear more resemblance to the myriad of causal judgments made in the real world, although slowed time perception may confer a depressive realism advantage in some experimental settings, this is unlikely to confer similar advantage in the real world.

## Conclusions

We set out to explore the role of time and context in causal learning, with levels of depression included as a moderator variable. Findings are not entirely consistent with either causal structure models or associative theories. Neither of these models can fully explain the absence of time effects on negative contingencies unless they make additional assumptions. For example, if contingency acts as a form of prior knowledge then the question of the psychological precedence of time over contingency becomes irrelevant because both time and contingency would exert their effect through prior knowledge. However, if mildly depressed participants data are considered to be representative of enhanced time effects through slowed time perception, then findings are more consistent with an associative model. These findings are also consistent with the idea that the crucial difference in causal learning, between those scoring low and high on a depression scale, is located in contextual learning.
