# Correction: Context and Time in Causal Learning: Contingency and Mood Dependent Effects

**DOI:** 10.1371/journal.pone.0114193

**Published:** 2014-11-18

**Authors:** The PLOS ONE Staff

## Notice of Republication

This article was republished on October 29, 2014, due to multiple instances of "*Δ*" being published as "?", as well as a formatting error in the “Contingency and time in causal learning” subsection of the Introduction. The publisher apologizes for this error. Please download the PDF again to view the corrected article. The originally published, uncorrected article and the republished, corrected article are provided here for reference.

## Supporting Information

File S1 Originally published, uncorrected article
(PDF)

File S2 Republished, corrected article
(PDF)
